# Comparative Analysis of Eight Mitogenomes of Bark Beetles and Their Phylogenetic Implications

**DOI:** 10.3390/insects12100949

**Published:** 2021-10-18

**Authors:** Huicong Du, Jiaxing Fang, Xia Shi, Sufang Zhang, Fu Liu, Chunmei Yu, Zhen Zhang, Xiangbo Kong

**Affiliations:** 1 Key Laboratory of Forest Protection of National Forestry and Grassland Administration, Research Institute of Forest Ecology, Environment and Protection, Chinese Academy of Forestry, Beijing 100091, China; dhc962@caf.ac.cn (H.D.); fjxinsect@163.com (J.F.); 18811771834@139.com (X.S.); Zhangsf@caf.ac.cn (S.Z.); liufu2006@163.com (F.L.); zhangzhen@caf.ac.cn (Z.Z.); 2 Qinghai Provincial Forest Diseases and Pest Control and Quarantine General Station, Xining 810007, China; qxshu@126.com

**Keywords:** bark beetle, Scolytinae, mitochondrial genome, tRNA, phylogeny, genetic distances

## Abstract

**Simple Summary:**

Many bark beetles are destructive pests in coniferous forests and cause extensive ecological and economic losses worldwide. Comparative studies of the structural characteristics of mitogenomes and phylogenetic relationships of bark beetles can improve our understanding of mitogenome evolution. In this study, we sequenced eight mitogenomes of bark beetles. Our results show that the use of start and stop codons, the abundance of amino acids, and the relative frequency of codon use are conserved among the eight bark beetles. Different regions of tRNA exhibit different degrees of conservatism. Together with the analysis of evolutionary rates and genetic distance among bark beetle species, our results reveal phylogenetic relationships among bark beetles of the subfamily Scolytinae.

**Abstract:**

Many bark beetles of the subfamily Scolytinae are the most economically important insect pests of coniferous forests worldwide. In this study, we sequenced the mitochondrial genomes of eight bark beetle species, including *Dendroctonus micans*, *Orthotomicus erosus*, *Polygraphus poligraphus*, *Dryocoetes hectographus*, *Ips nitidus*, *Ips typographus*, *Ips subelongatus*, and *Ips hauseri*, to examine their structural characteristics and determine their phylogenetic relationships. We also used previously published mitochondrial genome sequence data from other Scolytinae species to identify and localize the eight species studied within the bark beetle phylogeny. Their gene arrangement matched the presumed ancestral pattern of these bark beetles. Start and stop codon usage, amino acid abundance, and the relative codon usage frequencies were conserved among bark beetles. Genetic distances between species ranged from 0.037 to 0.418, and evolutionary rates of protein-coding genes ranged from 0.07 for *COI* to 0.69 for *ND2*. Our results shed light on the phylogenetic relationships and taxonomic status of several bark beetles in the subfamily Scolytinae and highlight the need for further sequencing analyses and taxonomic revisions in additional bark beetle species.

## 1. Introduction

Bark beetles (Coleoptera: Curculionidae: Scolytinae) feed mainly on the phloem and xylem of their hosts (e.g., pines, spruces, larches), severely affecting forest ecology and reducing forestry production [1,2]. Because they bore cryptically under the bark and into the wood, they are difficult to detect at the onset of the threat, resulting in many bark beetle species becoming established outside their original range [3]. In addition, international trade has led to the increasing establishment of exotics, including many invasive species from the subfamily Scolytinae [4]. A recent example is *Gnathotrichus materiarius* (Fitch, 1868) and *Cyclorhipidion bodoanum* (Reitter, 1913), which have been newly recorded as alien species for the British Isles [5]. Because bark beetles are both highly damaging and difficult to control, many researchers have studied the developmental trends of bark beetle species/populations using molecular markers [6,7,8,9,10]. The mitochondrial genome of insects, which is characterized by low molecular weight, simple structure, maternal inheritance, and rapid evolutionary rate [11,12,13,14,15], is widely used to study insect phylogeny and population inheritance [16,17,18]. With the development of DNA sequencing technology, the mitochondrial genomes of more and more insect species are being sequenced [19,20,21]. Nevertheless, data on the mitochondrial genomes of bark beetles remain limited. Cognato et al. [22] speculated that bark beetles in China may have high genetic diversity. In addition, taxonomic identification of some bark beetles is very important due to their pest status. Although thousands of species in the subfamily Scolytinae are not known to have economic impacts, they are ecologically important because they are the first decomposers of woody materials [23].

In this study, we sequenced and annotated the mitochondrial genomes of eight bark beetles, including *Dendroctonus micans* (Kugelann, 1794), *Orthotomicus erosus* (Wollaston, 1857), *Polygraphus poligraphus* (Linnaeus, 1758), *Dryocoetes hectographus* (Reitter, 1913), *Ips nitidus* (Eggers, 1933), *Ips typographus* (Linnaeus, 1758), *Ips subelongatus* (Motschulsky, 1860), and *Ips hauseri* (Reitter, 1894). By comparing these mitochondrial genomes, we can not only understand their structural characteristics, but also explore events in the evolutionary process of bark beetles that involve rearrangements and variations in mitochondrial genes. In addition, we determined the phylogenetic relationships between the eight species analyzed here and 18 other bark beetle species and 22 undetermined species from the subfamily Scolytinae whose mitochondrial genomes were sequenced. Our analysis provides new data for studying the phylogenetic relationships of some important bark beetles.

## 2. Materials and Methods

### 2.1. Sampling and DNA Extraction

Eight species of bark beetles, including *Dendroctonus micans*, *O. erosus*, *P. poligraphus*, *Dryocoetes hectographus*, *I. nitidus*, *I. typographus*, *I. subelongatus*, and *I. hauseri*, were collected in the field. Dr. Fang used a taxonomic search [24] to identify the species based on their body characteristics (e.g., teeth, body size, frontal tubercles, number of frontal hairs) along with the host tree species, and the detailed information is provided in Table 1. All collected specimens were stored in the Insect Museum of Chinese Academy of Forestry (Curator: Xiangbo Kong, Beijing, China). Live adult specimens were preserved in anhydrous ethanol and stored in a freezer at −20 °C. Total genomic DNA was extracted from pronotum and leg muscle tissue (five individuals per species) using a Wizard^®^ Genomic DNA Purification Kit (Promega Corporation, Madison, WI, USA) according to the manufacturer’s protocol. In conjunction with the NanoDrop 2000 Fluorospectrometer (Thermo Fisher Scientific, Wilmington, DE, USA), the PicoGreen^®^ assay (Thermo Fisher Scientific, Wilmington, DE, USA) was used to quantify dsDNA, and dsDNA integrity was determined by 1% agarose gel electrophoresis.

### 2.2. Mitogenome De Novo Sequencing and Assembly

Genomic DNA was fragmented to 400–500 bp using a Covaris^®^ M220 focused ultrasonicator (Covaris, Woburn, MA, USA). A 460 bp paired-end library was generated from each sample separately and finally sequenced using an Illumina HiSeq X Ten platform (Majorbio Bio-pharm Technology, Shanghai, China) to obtain 4 Gb of data. Reads with low sequencing quality were filtered out using Trimmomatic software (v0.36, http://www.usadellab.org/cms/?page=trimmomatic). Sequences were removed if they: contained splice fragments, had low mass values (Q < 25), or had lengths of low mass bases greater than half the total sequence length. The software Spades (Center for Algorithmic Biotechnology, St. Petersburg, Russia) [25] was used to assemble clean reads, and the assembly results were compared by mapping to obtain the longest segment. The MitoZ program (BGI-Shenzhen, Shenzhen, China) [26] was used to annotate the assembled genome, and the coding sequences (CDS), transfer RNA (tRNA), and ribosomal RNA (rRNA) were identified. The mitochondrial genome sequences of all eight species were deposited at GenBank (see Table 1 for deposit numbers).

### 2.3. Comparative Mitochontrial Genome Analysis

The nucleotide composition of the mitochondrial genome, including the content of A, T, C, and G bases, AT and GC skew, amino acid usage, and relative synonymous codon usage (RSCU) was calculated using MEGA 5.2.2 software (MEGA Software, Paris, France) [27]. Compositional skew was calculated using the formulas AT skew = (A − T)/(A + T) and GC skew = (G − C)/(G + C) [28]. tRNA genes were determined using the tRNAscan–SE Search Server (http://lowelab.ucsc.edu/tRNAscan-SE) (v2.0) and the MITOS [29] web server with invertebrate mitochondrial genetic codes [30].

### 2.4. Genetic Distance and Selection Pressure Analysis

To measure genetic distances among bark beetles of the subfamily Scolytinae, we used 26 mitogenomes, 8 of which were sequenced in our study and 18 of which were from the NCBI database to perform the corresponding analyses. Sequences were aligned using the Clustal X program and pairwise genetic distances were calculated using the MEGA v5.2.2 (MEGA Software, Paris, France) program based on Kimura 2-parameter model [31,32]. The software ClustalX was used to align the nucleic acid sequences of 13 protein-coding genes (PCGs). The nonsynonymous substitution rate (Ka), synonymous substitution rate (Ks), and Ka/Ks of the PCGs were determined using DnaSP v6.12.03 software [33].

### 2.5. Phylogenetic Analysis

To illustrate the phylogenetic relationships of the eight species of the subfamily Scolytinae in a broader evolutionary context, we constructed phylogenetic trees by analyzing the 50 mitochondrial genomes of the species of the subfamily Scolytinae (Supplementary Table S1), using *Sitophilus zeamais* (Motschulsky, 1855) and *Sitophilus oryzae* (Linnaeus, 1763) as outgroups [34]. PCGs, rRNA, and tRNA were aligned and concatenated in each mitochondrial genome using standard ClustalW parameters. Phylogenetic trees were constructed using the maximum likelihood method (ML) and Bayesian inference method (BI). The software MEGA v5.2 (MEGA software, Paris, France) was used to analyze the alternative models (GTR+G) and construct the tree ML. The confidence values of each branch node of the phylogenetic tree were bootstrapped with 1000 replicates [35]. The software MrBayes 3.2.2 [36] was used to construct the tree BI and MrMTgui software [37] to compare and analyze the alternative models of the sequences, and the optimal alternative model GTR+G was used to construct the evolutionary tree. The MCMC method was used to simulate 10,000,000 generations, sampling once every 1000 generations to ensure sampling independence. The first 25% of the simulations were discarded as burn-in. Stationarity was considered achieved when the average standard deviation of the split frequencies was less than 0.01 [38].

## 3. Results

### 3.1. Characteristics of the Mitochondrial Genome

The genome lengths for *O. erosus*, *Dryocoetes hectographus*, *P. poligraphus*, *Dendroctonus micans*, *I. hauseri*, *I. subelongatus*, *I. typographus* and *I. nitidus* were 16,753, 15,495, 15,586, 16,807, 15,516, 15,259, 15,376, and 15,359 bp, respectively. All mitochondrial genomes of each species consisted of thirteen PCGs, twenty-two tRNAs, and two rRNAs. Of these thirty-seven genes, fourteen genes were encoded by the N chain, including two rRNAs (*12SRNA* and *16SRNA*), four PCGs (*ND1*, *ND4*, *ND4L*, and *ND5*), and eight tRNAs (*trnV*, *trnL1*, *trnP*, *trnH*, *trnF*, *trnY*, *trnC*, and *trnQ*). The remaining twenty-three genes were encoded by the J chain, including nine PCGs and fourteen tRNAs. The structure of PCGs in the bark beetle mitogenome followed the same pattern as in *Iberobaenia* beetles [39], with the following sequence: *ND2*–*COI*–*COII*–*ATP8*–*ATP6*–*COIII* –*ND3*–*ND5*–*ND4*–*ND4L*–*ND6*–*CYTB*–*ND1* (Supplementary Tables S2–S9).

### 3.2. Nucleotide Composition and Condon Use

The AT content of the whole mitochondrial genome was above 70% in all eight species studied here, with *Dendroctonus micans* having the highest value (75.72%) and *O. erosus* the lowest (70.69%). In the sequences of PCGs, tRNA, and rRNA, the AT content was also significantly higher than that of CG, with the AT content being highest in rRNA and lowest in PCGs (Supplementary Table S10). All eight Scolytinae mitogenomes showed a positive AT skew (0.335 to 0.414) and a negative GC skew (−0.2722 to −0.2421).

Except for the *ND1* gene in *Ips* bark beetle which used TTG, PCGs of all species used ATN as the start codon (Supplementary Tables S2–S9). The use of stop codons also varied widely within and between species. In this study, we found two types of stop codons: the complete stop codons TAA and TAG and the incomplete stop codon T-. The *ND1* and *ND5* genes of *I. hauseri* used the incomplete stop codon T- (Supplementary Table S6), while the complete stop codon TAG was mainly found in the *ATP8*, *ND4L*, *ND3*, *ND5*, *ND1*, and *CYTB* genes of all eight species, while other genes used TAA as the stop codon.

### 3.3. Comparison of Codon Usage in Protein-Coding Genes and tRNA Secondary Structure

**Figure 1:**
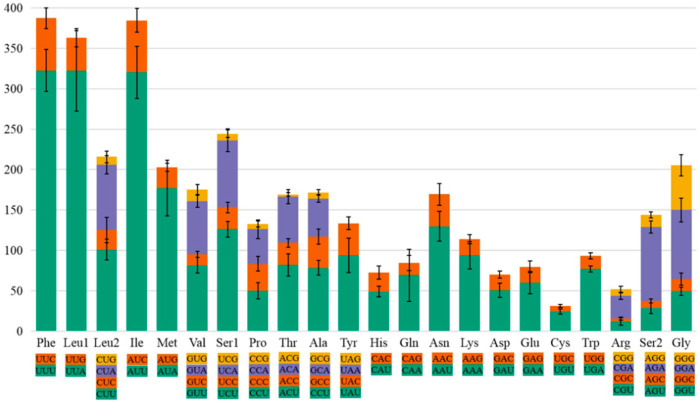
The use of amino acids of the protein-coding genes of the mitogenome in eight bark beetles. Codon families are indicated on the *x*-axis and amino acid numbers on the *y*-axis. The error bars represent the standard error.

**Figure 2:**
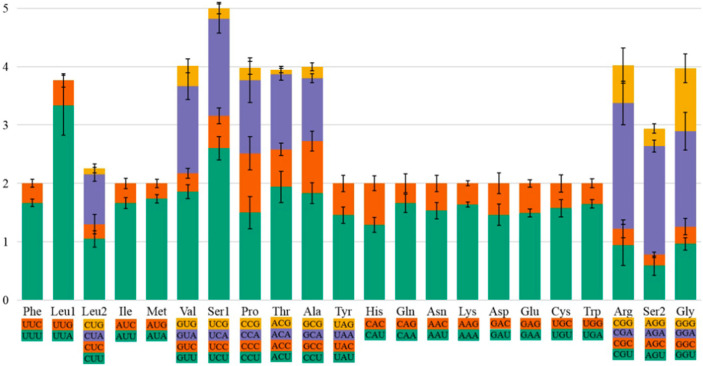
The relative synonymous codon usage (RSCU) of the protein-coding genes of the mitogenome in eight bark beetles. Codon families are indicated on the *x*-axis and RSCU values on the *y*-axis. The error bars represent the standard error.

Analysis of the amino acid content and relative codon usage frequency in the PCGs of the eight species revealed the presence of all possible synonymous codons of the 22 amino acids (Figure 1). Among these, Phe, Leu1, and Ile were the most abundant with frequencies greater than 300, and Cys was the least abundant with frequencies less than 50. Analysis of the relative use of synonymous codons showed that RSCU was highest for NNA and NNU codons, generally greater than 1, and lowest for NNG and NNC codons (Figure 2). Codons with relatively high G and C content were rare, confirming a finding in other insect species. Overall, UUA, UCU, and GUU were the three most common relatively synonymous codons.

The tRNA length in the eight species analyzed here ranged from 62 bp to 72 bp. Most base pairs in the stem region conformed to the Watson–Crick pairing principle, and a few followed the G–U vibrational pairing principle. There were 166 G–U pairs, with *I. hauseri* having the most mismatched pairs and *Dendroctonus micans* having the fewest. In our data, mismatched bases in the secondary structure of tRNA occurred mainly in the amino acid acceptor arm and least frequently in the TΨC arm (Table 2). Thus, the different regions showed different degrees of conservatism, with the TΨC arm being the most conservative. Differences in tRNA secondary structure were small among species belonging to the same genus or closely related species.

### 3.4. Phylogenetic Analysis

**Figure 3:**
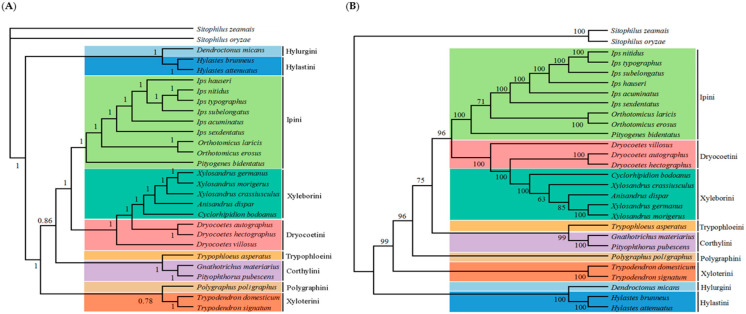
Phylogenetic tree inferred from mitogenomes of the subfamily Scolytinae using Bayesian inference method (**A**) and maximum likelihood method (**B**). Values at nodes indicate Bayesian posterior probabilities and bootstrap values for the BI and ML trees, respectively. Clades with different colors indicate different tribe.

Bayesian phylogenetic trees constructed from the complete mitochondrial genome had high confidence values, and the monophyly of the tribe and genera of the species studied was well supported (Figure 3A). The phylogenetic relationships of the subfamily Scolytinae by tribe were determined as follows: (((Ipini + (Xyleborini + Dryocoetini)) + (Trypophloeini + Corthylini)) + (Polygraphini + Xyloterini)) + (Hylurgini + Hylastini)). The topologies of the phylogenetic trees constructed based on the methods of ML are similar to those of the BI tree (Figure 3B), and the monophyly of the tribe and most genera was also well supported in the ML tree. However, the phylogenetic relationships between some species are still confusing. For example, both the ML tree and the BI tree show that the genus *Dryocoetes* is poorly defined due to the position of the species *Dryocoetes villosus* (Fabricius, 1792) in the phylogenetic tree. In addition, *Xylosandrus crassiusculus* (Motschulsky, 1866) and *Anisandrus dispar* (Fabricius, 1792) have different positions in the ML tree and BI tree. The genus *Xylosandrus* is monophyletic in the BI tree but not in the ML tree. We also constructed the phylogenetic tree for the undetermined species of the subfamily Scolytinae using the data from the NCBI database (Supplementary Figure S9), which showed that eight species are classified in Xyleborini (*Xyleborus* BMNH1040067; Scolytinae BMNH 1039855; Scolytinae BMNH1274287; Scolytinae BMNH1040341; Scolytinae BMNH1039965; Scolytinae BMNH1043104; Scolytinae BMNH1040075; Scolytinae BMNH1040174), eight species are classified in Trypophloeini (Scolytinae BMNH1040327; Scolytinae BMNH1040118; Scolytinae BMNH1040002; Scolytinae BMNH1039905; *Hypothenemus* BMNH1040235; *Hypothenemus* BMNH1039837; *Hypothenemus* BMNH1040003; *Hypothenemus* BMNH1039866), three species are classified in Corthylini (Scolytinae BMNH1040351; Scolytinae BMNH1039994; Scolytinae BMNH1039990), one species in Ipini (Scolytinae BMNH1040265), and two species cannot be located (Scolytinae BMNH10403133; Scolytinae BMNH1040331).

### 3.5. Analysis of the Genetic Distance and the Evolutionary Rate

**Figure 4:**
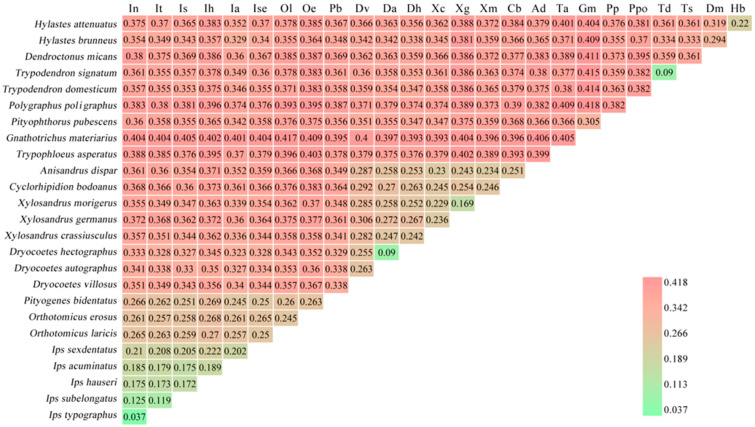
Genetic distance of bark beetles in Scolytinae. Species names on the horizontal axis are abbreviated by the first letter of the two words of the species name, and one letter is added to the second word of the species name if the two letters are the same.

Genetic distances between sequenced bark beetles based on the whole mitogenome supported their phylogenetic relationships. The distances between species within a genus were shorter than the distances between genera. The shortest distance, and thus the closest genetic relationship, was between *I. typographus* and *I. nitidus* (0.037), whereas the greatest distance was between *P. poligraphus* and *G. materiarius* (0.418) (Figure 4). Among the bark beetles of the Scolytinae, most of the genetic distances between species were greater than 0.1, except for the distances between *Dryocoetes hectographus* and *Dryocoetes autographus*, *Trypodendron signatum* (Fabricius, 1792) and *Trypodendron domesticum* (Linnaeus, 1758), and *I. typographus* and *I. nitidus*, suggesting that they are sibling species.

**Figure 5:**
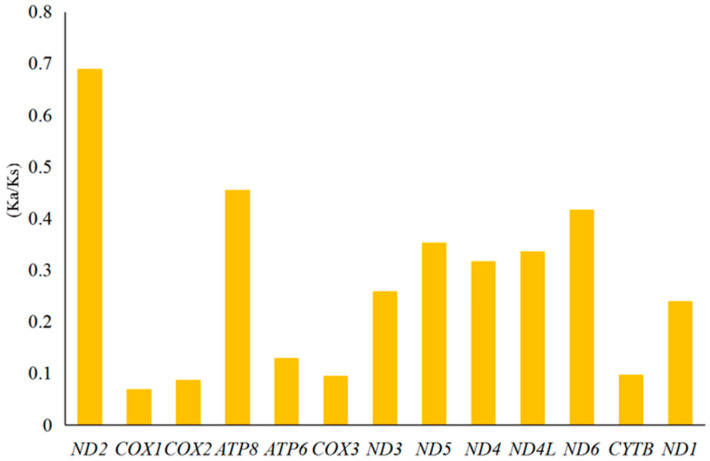
Evolutionary rates of PCGs in bark beetle mitogenomes in Scolytinae. The *y*-axis indicates the Ka/Ks ratio of mitochondrial PCGs and the *x*-axis indicates 13 genes encoding proteins in mitochondria.

The Ka/Ks ratio, a diagnostic statistical method for detecting molecular adaptations, is used to estimate the evolutionary rate among insects. Evolutionary rates of PCGs among Scolytinae species, as measured by rates of nonsynonymous (Ka) and synonymous (Ks) substitutions and Ka/Ks ratio, are shown in Figure 5. Average Ka/Ks ratios were consistently less than 1 and ranged from 0.07 for *COI* to 0.69 for *ND2*, indicating that these mitogenomes evolve under purifying selection. All mitochondrial PCGs could be used to examine phylogenetic relationships within the Scolytinae.

## 4. Discussion

In this study, we performed a comparative mitogenome analysis and revealed the conservatism of the eight bark beetle mitogenomes. The sequences of the control region of mitogenome were not assembled because their distribution and length are uncertain. Most of the sequences in the control region are located between the *rrnS* and *trnI* genes, and some of them are divided into two segments by the *trnI* gene [40,41]. The structure of PCGs in the bark beetle mitogenome followed the same pattern as in Iberobaenia beetles; gene rearrangement events, especially those related to PCGs, are rare in mitogenomes of Coleoptera [42]. Bark beetles exhibit the typical AT-biased composition of insect mitogenomes [43,44]. In them, AT content was significantly lower in PCGs and significantly higher in rRNA and tRNA, which might be due to PCGs being more functionally restricted than other regions. The biological reasons for such AT-biased composition have been extensively studied [45,46], and it has been hypothesized that AT-biased composition is more energy efficient [47,48]. We have also found that base shifts in the same region of the mitogenome are similar between species, with PCGs showing a strong preference for T and C, rRNA for T and G, and tRNA for A and G. Although the mechanisms for such skewed composition are complex, most hypotheses suggest that this phenomenon is due to mutation and selection pressure [49]. In insect mitogenomes, the extent of GC-biased composition appears to be related to gene replication and selection pressure during transcription [45], which is not consistent with the direction of mutations [49,50]. Regarding the use of start and stop codons in PCGs, PCGs of most species used ATN as the start codon. The start codons for *ND1* are often non-standard in holometabolous insects, with the non-standard TTG being used in almost half of the known mitogenomes of species in the Curculionidae [51]. Stop codons include TAA, TAG, and T-, and the incomplete stop codon (T-) can become the complete stop codon by polyadenylation during posttranscriptional mRNA processing TAA [52], which is also common in the insect mitogenome [53]. Moreover, the start and stop codons also had the property of being AT-biased.

According to the secondary structure model of mitochondrial tRNA genes proposed by Kumazawa and Nishida [54], tRNA sequences can be arranged in stem-loop structures. With the exception of the tRNASer (AGN), which lacks the DHU (dihydrouridine) arm, all others are classical cloverleaf structures. In addition, we found that base mismatches are abundant in the secondary structure of bark beetles, which is common in the secondary structure of insect tRNA. For example, the ghost moth *Thitarodes yunnanensis* Yang has 18 mismatches [55], while the nymphalid butterfly *Parathyma sulpitia* Cramer has 22 mismatches [56]. Yokobori and Paabo [57] speculated that these mismatches can be explained by the lack of recombination in the insect mitogenome, making such mutations difficult to remove. These mismatches occurred mainly on the amino acid acceptor arm of the secondary structure of eight bark beetles, and least on the TΨC arm. The closer the relationship, the smaller the structural difference and the more similar the number of mismatches. This result, combined with the uneven distribution of mismatched bases in different tRNA regions, highlights the need for a large-scale comparison of tRNA secondary structure between species. Such an analysis could help reveal the evolutionary pathways of insect mitochondrial tRNA and the functions of individual regions and base mutations, providing a basis for further research into the evolution of mitogenome structure.

The constructed phylogenetic trees strongly supported the monophyly of the tribe and most of the genera in our analysis. However, the position of *Dryocoetes villosus* in the trees of ML and BI is different, which needs to be redefined and revised. Jordal et al. [58] also found that the genus *Dryocoetes* is not monophyletic by examining the 28S gene, which cannot be separated from the genus *Taphrorychus* in the phylogenetic tree. The genus *Xylosandrus* currently contains species with highly variable morphology, some of which resemble those of other genera, which has led to confusion regarding the generic boundaries of *Xylosandrus* and its relationship to and separation from the genus *Anisandrus* [59]. Therefore, many scholars have made great efforts to classify this genus [60,61,62,63]. For example, Dole and Cognato have recently revised *Xylosandrus* [64] and preliminary phylogenies suggest that *Xylosandrus* is monophyletic [61]. In addition, we have also investigated the phylogenetic relationships of some unidentified species in the subfamily Scolytinae using data from the NCBI database. However, these species are not placed in a well supported lineage, and much information and mitochondrial data are needed to define their phylogenetic relationships. We also analyzed the genetic distances among bark beetles, which supports the phylogenetic relationships of the studied species. An analysis of vertebrate and invertebrate *COI* gene sequences by Hebert et al. [65] revealed an average interspecific difference in genetic distance of approximately 0.113 in 98% of species. Within *Ips* species, Chang et al. [66] found an interspecific genetic distance between 0.056 and 0.431, similar to our results.

In general, we have described the characteristics of the mitogenomes of eight species from the subfamily Scolytinae and performed a comparative analysis of the sequence of gene arrangement, nucleotide composition, use of PCGs codons, tRNA secondary structure, genetic distance, and evolutionary rate among bark beetle species. The typical structural characteristics and conservatism of the bark beetle mitogenome suggest that it can be used to clarify phylogenetic relationships at higher taxonomic levels. Based on the mitogenome data, the constructed phylogenetic relationship among species of Scolytinae has produced an in-depth study of the genetic evolution of bark beetles. However, the sequenced species are still quite few, which directly limits a definitive species delimitation to an undisputed lineage in bark beetles.

## Figures and Tables

**Table 1:** Sampling information for eight bark beetles.

Species	Location (District, Province)	Latitude (°)	Longitude (°)	GenBank Numbers
*Dendroctonus micans*	Maixiu, Qinghai	35.27	101.91	MZ768861
*Polygraphus poligraphus*	Qilian, Qinghai	38.18	100.32	OK110248
*Dryocoetes hectographus*	Qilian, Qinghai	38.18	100.32	MZ766132
*Orthotomicus erosus*	Yuxi, Yunnan	24.13	102.10	MZ823388
*Ips typographus*	Habahe, Xinjiang	48.47	86.68	MZ766131
*Ips subelongatus*	Yichun, Heilongjiang	48.65	126.63	MZ766130
*Ips hauseri*	Tianshan, Xinjiang	43.18	82.85	MZ768860
*Ips nitidus*	Maixiu, Qinghai	35.26	101.89	MZ748471

**Table 2:** Comparison of tRNA secondary structure mismatches in eight bark beetles.

Species	Amino Acid Acceptor Arm	DHU Arm	TΨC Arm	Anticodon Arm	Sum
*Ips nitidus*	13	7	1	3	24
*Ips subelongatus*	13	7	2	1	23
*Ips hauseri*	15	7	3	3	28
*Ips typographus*	10	8	1	3	22
*Orthotomicus erosus*	5	4	2	3	14
*Dryocoetes hectographus*	9	6	1	5	21
*Polygraphus poligraphus*	10	4	4	4	22
*Dendroctonus micans*	6	4	1	5	12
Mean	10	6	2	3	21

## Data Availability

All data in this article are deposited in GenBank with the accession numbers listed in Table 1.

## Supplementary Materials

The following are available online at https://www.mdpi.com/article/10.3390/insects12100949/s1, Table S1: The list of sample information of bark beetles. Table S2: Annotation of the mitogenome of *Orthotomicus erosus*. Table S3: Annotation of the mitogenome of *Dryocoetes hectographus*. Table S4: Annotation of the mitogenome of *Polygraphus poligraphus*. Table S5: Annotation of the mitogenome of *Dendroctonus micans*. Table S6: Annotation of the mitogenome of *Ips hauseri*. Table S7: Annotation of the complete mitogenome of *Ips subelongatus*. Table S8: Annotation of the mitogenome of *Ips typographus*. Table S9: Annotation of the mitogenome of *Ips nitidus*. Table S10. Investigation of nucleotide composition in the mitochondrial genomes of eight bark beetles. Figure S1: Secondary structures for tRNA genes from the mtDNA of *Dryocoetes hectographus*. Figure S2: Secondary structures for tRNA genes from the mtDNA of *Orthotomicus erosus*. Figure S3: Secondary structures for tRNA genes from the mtDNA of *Ips typographus*. Figure S4: Secondary structures for tRNA genes from the mtDNA of *Ips nitidus*. Figure S5: Secondary structures for tRNA genes from the mtDNA of *Ips hauseri*. Figure S6: Secondary structures for tRNA genes from the mtDNA of *Ips subelongatus*. Figure S7: Secondary structures for tRNA genes from the mtDNA of *Dendroctonus micans*. Figure S8: Secondary structures for tRNA genes from the mtDNA of *Polygraphus poligraphus*. Figure S9: Phylogenetic tree inferred from mitogenomes of subfamily Scolytinae using Bayesian inference method (A) and maximum likelihood method (B). Values at nodes indicate Bayesian posterior probabilities and bootstrap values for the BI and ML trees, respectively.

## References

[1] Fang J.X., Liu M., Zhang S.F., Liu F., Zhang Z., Zhang Q.H., Kong X.B. (2020). Chemical signal interactions of the bark beetle with fungal symbionts, and host/non–host trees. J. Exp. Bot..

[2] Shi X., Zhang S.F., Liu F., Xu F.Y., Zhang F.B., Guo X.B., Zhang Z., Kong X.B. (2020). SEM analysis of sensilla on the mouthparts and antennae of Asian larch bark beetle *Ips Subelongatus*. Micron.

[3] Johnson A.J., Hulcr J., Knížek M., Atkinson T.H., Mandelshtam M.Y., Smith S.M., Cognato A.I., Park S., Li Y., Jordal B.H. (2020). Revision of the Bark Beetle Genera Within the Former Cryphalini (Curculionidae: Scolytinae). Insect Syst. Diver..

[4] Hughes M.A., Riggins J.J., Koch F.H., Cognato A.I., Anderson C., Formby J.P., Dreaden T.J., Ploetz R.C., Smith J.A. (2017). No rest for the laurels: Symbiotic invaders cause unprecedented damage to southern USA forests. Biol. Invasions.

[5] Inward D.J.G. (2020). Three new species of ambrosia beetles established in Great Britain illustrate unresolved risks from imported wood. J. Pest Sci..

[6] Biedermann P.H.W., Müller J., Grégoire J.C., Gruppe A., Hagge J., Hammerbacher A., Hofstetter R.W., Kandasamy D., Kolarik M., Kostovcik M. (2019). Bark beetle population dynamics in the Anthropocene: Challenges and solution. Trends Ecol. Evol..

[7] Lv F., Yang W.Y., Chen Z.T., Xu Q., Zhou Y.J., Du Y.Z. (2017). Three partial mitochondrial genomes from *Ips* (Coleoptera: Cruculionidae, Scolytinae) contribute to the phylogeny of Scolytinae. J. Asia-Pac. Entomol..

[8] Avtzis D.N., Lakatos F., Gallego D., Pernek M., Faccoli M., Wegensteiner R., Stauffer C. (2019). Shallow Genetic Structure among the European Populations of the Six-Toothed Bark Beetle *Ips sexdentatus* (Coleoptera, Curculionidae, Scolytinae). Forests.

[9] Pistone D., Gohli J., Jordal B.H. (2017). Molecular phylogeny of bark and ambrosia beetles (Curculionidae: Scolytinae) based on 18 molecular markers. Syst. Entomol..

[10] Jordal B.H., Kaidel J. (2016). Phylogenetic analysis of Micracidini bark beetles (Coleoptera: Curculionidae) demonstrates a single trans-Atlantic disjunction and inclusion of Cactopinus in the New World clade. Can. Entomol..

[11] Li X.Y., Yan L.P., Pape T., Gao Y.Y., Zhang D. (2020). Evolutionary insight into bot flies (Insecta: Diptera: Oestridae) from comparative analysis of the mitochondrial genomes. Int. J. Biol. Macromol..

[12] Song F., Li H., Liu G.H., Wang W., James P., Colwell D.D., Tran A., Gong S., Cai W., Shao R. (2019). Mitochondrial genome fragmentation unites the parasitic lice of Eutherian mammals. Syst. Biol..

[13] Liu Y., Song F., Jiang P., Wilson J.J., Cai W., Li H. (2018). Compositional heterogeneity in true bug mitochondrial phylogenomics. Mol. Phylogenet. Evol..

[14] Du H.C., Wang Y., Fang J.X., Zhang Z.Y., Zhang S.F., Liu F., Zhang Z., Kong X.B. (2019). Sequencing and analysis of the complete mitochondrial genome of *Dendrolimus punctatus* (Lepidoptera: Lasiocampidae). Sci. Silvae Sin..

[15] Du H.C., Liu M., Zhang S.F., Liu F., Zhang Z., Kong X.B. (2020). Lineage divergence of *Dendrolimus punctatus* in Southern China based on mitochondrial genome. Front. Genet..

[16] Boore J.L. (1999). Animal mitochondrial genomes. Nucleic Acids Res..

[17] Curole J.P., Kocher T.D. (1999). Mitogenomics: Digging deeper with complete mitochondrial genomes. Trends Ecol. Evol..

[18] Du Z.Y., Hasegawa H., Cooley J.R., Simon C., Yoshimura J., Cai W.Z., Li H. (2019). Mitochondrial genomics reveals shared phylogeographic patterns and demographic history among three periodical cicada species groups. Mol. Biol. Evol..

[19] Sharma A., Siva C., Ali S., Sahoo P.K., Nath R., Laskar M.A., Sarma D. (2020). The complete mitochondrial genome of the medicinal fish, *Cyprinion semiplotum*: Insight into its structural features and phylogenetic implications. Int. J. Biol. Macromol..

[20] Chen Z., Liu Y.Q., Wu Y.F., Song F., Cai W.Z., Li H. (2020). Novel tRNA gene rearrangements in the mitochondrial genome of *Camarochiloides weiweii* (Hemiptera: Pachynomidae). Int. J. Biol. Macromol..

[21] Li R., Zhang W., Ma Z.X., Zhou C.F. (2020). Novel gene rearrangement pattern in the mitochondrial genomes of *Torleya mikhaili* and *Cincticostella fusca* (Ephemeroptera: Ephemerellidae). Int. J. Biol. Macromol..

[22] Cognato A.I., Sperling F.A.H. (2000). Phylogeny of *Ips* DeGeer species (Coleoptera: Scolytidae) inferred from mitochondrial cytochrome oxidase I DNA sequence. Mol. Phylogenet. Evol..

[23] Ramirez–Rios V., Franco–Sierra N.D., Alvarez J.C., Saldamando–Benjumea C.I., Villanueva–Mejia D.F. (2016). Mitochondrial genome characterization of *Tecia solanivora* (Lepidoptera: Gelechiidae) and its phylogenetic relationship with other lepidopteran insects. Gene.

[24] Huang F.S., Lu J. (2015). The Classification Outline of Scolytidae from China.

[25] Dmitry A., Anton K., McLean J.S., Pevzner P.A. (2016). HYBRIDSPADES: An algorithm for hybrid assembly of short and long reads. Bioinformatics.

[26] Meng G., Li Y., Yang C., Liu S. (2019). MitoZ: A toolkit for animal mitochondrial genome assembly, annotation and visualization. Nucleic Acids Res..

[27] Tamura K. (2011). MEGA5: Molecular evolutionary genetics analysis using maximum likelihood, evolutionary distance, and maximum parsimony methods. Mol. Biol. Evol..

[28] Perna N.T., Kocher T.D. (1995). Patterns of nucleotide composition at fourfold degenerate sites of animal mitochondrial genomes. J. Mol. Evol..

[29] Bernt M., Donath A., Jühling F., Externbrink F., Florentz C., Fritzsch G., Pütz J., Middendorf M., Stadler P.F. (2013). MITOS: Improved de novo metazoan mitochondrial genome annotation. Mol. Phylogenet. Evol..

[30] Lowe T.M., Chan P.P. (2016). tRNAscan–SE On–line: Integrating search and context for analysis of transfer RNA genes. Nucleic Acids Res..

[31] Tamura K., Stecher G., Peterson D., Filipski A., Kumar S. (2013). MEGA6: Molecular evolutionary genetics analysis version 6.0. Mol. Biol. Evol..

[32] Kimura M. (1980). A simple method for estimating evolutionary rates of base substitutions through comparative studies of nucleotide sequences. J. Mol. Evol..

[33] Rozas J., Ferrer–Mata A., Sánchez–DelBarrio J.C., Guirao–Rico S., Librado P., Ramos–Onsins S.E., Sánchez–Gracia A. (2017). DnaSP 6: DNA sequence polymorphism analysis of large data sets. Mol. Biol. Evol..

[34] Ojo J.A., Valero M.C., Sun W., Coates B.S., Omoloye A.A., Pittendrigh B.R. (2016). Comparision of full mitochondrial genomes for the rice weevil, *Sitophilus oryzae* and the maize weevil, *Sitophilus zeamais* (Coleoptera: Curculionidae). Agri Gene.

[35] Guindon S., Gascuel O. (2003). A simple, fast, and accurate algorithm to estimate large phylogenies by maximum likelihood. Syst. Biol..

[36] Ronquist F., Huelsenbeck J.P. (2003). MrBayes 3: Bayesian phylogenetic inference under mixed models. Bioinformatics.

[37] Clary D.O., Wolstenholme D.R. (1985). The ribosomal RNA genes of Drosophila mitochondrial DNA. Nucleic Acids Res..

[38] Huelsenbeck J.P., Ronquist F., Nielsen R., Bollback J.P. (2001). Bayesian inference of phylogeny and its impact on evolutionary biology. Science.

[39] Carmelo A., Paula A., Benjamin L., Robin K., Ladislav B., Alfried P. (2016). The mitochondrial genome of iberobaenia (Coleoptera: Iberobaeniidae): First rearrangement of protein-coding genes in the beetles. Mitochondrial DNA Part A.

[40] Boyce T.M., Zwick M.E., Aquadro C.F. (1989). Mitochondrial DNA in the bark weevils: Size, structure and heteroplasmy. Genetics.

[41] Zhang F., Hong B., Wang Y.Z., Li Y.M., Chen Z.J. (2019). Sequencing and phylogenetic analysis of the comple mitochondrial genome of *Scythropus yasumatsui* (Coleoptera: Curculionidae). Acta Entomol. Sin..

[42] Timmermans M.J.T.N., Vogler A.P. (2013). Phylogenetically informative rearrangements in mitochondrial genomes of Coleoptera, and monophyly of aquatic elateriform beetles (Dryopoidea). Mol. Phylogenet. Evol..

[43] Bian D., Ye W.T., Dai M., Lu Z.T., Li M.X., Fang Y.L., Qu J.W., Su W.J., Li F.C., Sun H.N. (2020). Phylogenetic relationships of Limacodidae and insights into the higher phylogeny of Lepidoptera. Int. J. Biol. Macromol..

[44] Chen L.P., Zheng F.Y., Bai J., Wang J.M., Lv C.Y., Li X., Zhi Y.C., Li X.J. (2020). Comparative analysis of mitogenomes among six species of grasshoppers (Orthoptera: Acridoidea: Catantopidae) and their phylogenetic implications in wing–type evolution. Int. J. Biol. Macromol..

[45] Foerstner K.U., Mering C.V., Hooper S.D., Bork P. (2005). Environments shape the nucleotide composition of genomes. EMBO Rep..

[46] Chen W.H., Lu G., Bork P., Hu S., Lercher M.J. (2016). Energy effificiency trade–offs drive nucleotide usage in transcribed regions. Nat. Commun..

[47] Rocha E.P.C., Danchin A. (2002). Base composition bias might result from competition for metabolic resources. Trends Genet..

[48] Hassanin A., Leger N., Deutsch J. (2005). Evidence for multiple reversals of asymmetric mutational constraints during the evolution of the mitochondrial genome of metazoa, and consequences for phylogenetic inferences. Syst. Biol..

[49] Bogenhagen D.F., Clayton D.A. (2003). The mitochondrial DNA replication bubble has not burst. Trends Biochem. Sci..

[50] Brown T.A., Cecconi C., Tkachuk A.N., Bustamante C., Clayton D.A. (2005). Replication of mitochondrial DNA occurs by strand displacement with alternative light—strand origins, not via a strand—coupled mechanism. Gene. Dev..

[51] Chen Y., Luo C.B., Li Y.Q., Yang Y.J. (2019). Mitochondrial genome characteristics and phylogenetic analysis of the Curculionidae. J. Environ. Entomol..

[52] Anderson S., Bankier A.T., Barrell B.G., Bruijn M.H.L.D., Coulson A.R., Drouin J., Eperon I.C., Nierlich D.P., Roe B.A., Sanger F. (1981). Sequence and organization of the human mitochondrial genome. Nature.

[53] Liu Y., Cui Z. (2009). The complete mitochondrial genome sequence of the cutlassfish *Trichiurus japonicus* (Perciformes: Trichiuridae): Genome characterization and phylogenetic considerations. Mar. Genom..

[54] Kumazawa Y., Nishida M. (1993). Sequence evolution of mitochondrial tRNA genes and deep–branch animal phylogenetics. J. Mol. Evol..

[55] Cao Y.Q., Ma C., Chen J.Y., Yang D.R. (2012). The complete mitochondrial genomes of two ghost moths, *Thitarodes renzhiensis* and *Thitarodes yunnanensis*: The ancestral gene arrangement in Lepidoptera. BMC Genom..

[56] Tian L.L., Sun X.Y., Chen M., Gai Y.H., Hao J.S., Yang Q. (2012). Complete mitochondrial genome of the five–dot sergeant *Parathyma sulpitia* (Nymphalidae: Limenitidinae) and its phylogenetic implications. Zool. Res..

[57] Yokobori S., Paabo S. (1995). Transfer RNA editing in land snail mitochondria. Proc. Natl. Acad. Sci. USA.

[58] Jordal B.H., Kambestad M. (2014). DNA barcoding of bark and ambrosia beetles reveals excessive NUMTs and consistent east-west divergence across Palearctic forests. Mol. Ecol. Resour..

[59] Dole S.A., Jordal B.H., Cognato A.I. (2010). Polyphyly of *Xylosandrus* Reitter inferred from nuclear and mitochondrial genes (Coleoptera: Curculionidae: Scolytinae). Mol. Phylogenet. Evol..

[60] Cognato A.I., Smith S.M., Beaver R.A. (2020). Two new genera of Oriental xyleborine ambrosia beetles (Coleoptera, Curculionidae: Scolytinae). Zootaxa.

[61] Smith S.M., Beaver R.A., Cognato A.I. (2020). A monograph of the Xyleborini (Coleoptera, Curculionidae, Scolytinae) of the Indochinese Peninsula (except Malaysia) and China. ZooKeys.

[62] Skelton J., Johnson A.J., Jusino M.A., Bateman C.C., Li Y., Hulcr J. (2019). A selective fungal transport organ (mycangium) maintains coarse phylogenetic congruence between fungus-farming ambrosia beetles and their symbionts. Proc. R. Soc. B.

[63] Johnson A.J., McKenna D.D., Jordal B.H., Cognato A.I., Smith S.M., Lemmon A.R., Lemmon E.M., Hulcr J. (2018). Phylogenomics clarifies repeated evolutionary origins of in breeding and fungus farming in bark beetles (Curculionidar, Scolytinae). Mol. Phylogent. Evol..

[64] Dole S.A., Cognato A.I. (2010). Revision of *Xylosandrus* Reitter (Curculionidae: Scolytinae). Proc. Calif. Sci..

[65] Hebert P.D., Cywinska A., Ball S.L., Waard J.R. (2003). Biological identifications through DNA barcodes. Proc. R. Soc. Lond. B Biol. Sci..

[66] Chang H., Hao D.J., Xiao R.T., Liu Y., Qian L., An Y.L., Yang X.J. (2012). DNA barcoding based on the mitochondrial *COI* gene sequences for *Ips* species (Coleoptera: Scolytinae). Acta Entomol. Sin..

