# Supplementary material for: Comparative Analysis of Eight Mitogenomes of Bark Beetles and Their Phylogenetic Implications
Source: Insects. 2021 Oct 18;12(10):949. doi: 10.3390/insects12100949 (PMC8538572; doi:10.3390/insects12100949)
Supplement: Supplementary file 1 [file insects-12-00949-s001.zip › insects-1395895-supplementary.pdf]

Table S1. The list of sample information of bark beetles.

Species	Accession no.	Species	Accession no.
Hylates brunneus	NC_036262.1	Scolytinae BMNH 1039855	X035164.1
Hylates attenuatus	NC_036290.1	Scolytinae BMNH1274287	KT696206.1
Pityophthorus pubescens	NC_036288.1	Scolytinae BMNH1040341	KX035192.1
Pityogenes bidentatus	NC_036289.1	Scolytinae BMNH1039965	KX035170.1
Ips acuminatus	MK988441	Scolytinae BMNH1043104	KX035197.1
Ips sexdentatus	NC_036281.1	Scolytinae BMNH1040075	KX035181.1
Orthotomicus laricis	NC_036291.1	Scolytinae BMNH1040174	KX035185.1
Dryocoetes autographus	NC_036287.1	Scolytinae BMNH1040327	KX035189.1
Dryocoetes villosus	NC_036282.1	Scolytinae BMNH1040118	KX035183.1
Xylosandrus morigerus	NC_036283.1	Scolytinae BMNH1040002	KX035174.1
Xylosandrus germanus	NC_036280.1	Scolytinae BMNH1039905	KX035168.1
Xylosandrus crassiusculus	NC_036284.1	*Hypothenenmus* BMNH1040235	KX035186.1
Anisandrus dispar	NC_036293.1	*Hypothenenmus* BMNH1039837	KX035163.1
Cyclorhipidion bodoanum	NC_036295	*Hypothenenmus* BMNH1040003	KX035175.1
Gnathotrichus materiarius	NC_036294.1	*Hypothenenmus* BMNH1039866	KX035165.1
Trypophloeus asperatus	NC_036285.1	Scolytinae BMNH1040351	KX035193.1
Trypodendron signatum	NC_036292.1	Scolytinae BMNH1039994	KX035172.1
Trypodendron domesticum	NC_036286.1	Scolytinae BMNH1039990	KX035171.1
Sitophilus oryzae	KX373615	Scolytinae BMNH1040265	KX035187.1
Sitophilus zeamais	KX373614	Scolytinae BMNH10403133	KX035199.1
Xyleborus BMNH1040067	KX035179.1	Scolytinae BMNH1040331	KX035190.1

Table S2. Annotation of the mitogenome of *Orthotomicus erosus.*

Gene	Direction	Start position	Stop position	Length	Anticodon	Start Codon	Stop Codon	IGS
trnQ	R	1	70	70	CAT			
trnM	F	116	183	68	TTG			45
ND2	F	202	1191	990		ATT	TAA	18
trnW	F	1211	1278	68	TCA			19
trnC	R	1298	1363	66	GCA			19
trnY	R	1394	1459	66	GTA			30
COI	F	1506	3050	1545		ATA	TAA	46
trnL2	F	3046	3112	67	TAA			-5
COII	F	3068	3796	729		ATG	TAA	-45
trnK	F	3804	3874	71	CTT			7
trnD	F	3887	3954	68	GTC			12
ATP8	F	3964	4125	162		ATA	TAG	9
ATP6	F	4119	4802	684		ATG	TAA	-7
COIII	F	4807	5601	795		ATG	TAA	4
trnG	F	5639	5704	66	TCC			37
ND3	F	5705	6058	354		ATT	TAG	0
trnA	F	6106	6173	68	TGC			47
trnR	F	6184	6249	66	TCG			10
trnN	F	6325	6389	65	GTT			75
trnS1	F	6390	6456	67	GCT			0
trnE	F	6457	6523	67	TTC			0
trnF	R	6548	6616	69	GAA			24
ND5	R	6617	8326	1710		ATT	TAA	0
trnH	R	8342	8406	65	GTG			15
ND4	R	8469	9815	1347		ATG	TAA	62
ND4L	R	9809	10096	288		ATG	TAG	-7
trnT	F	10114	10180	67	TGT			17
trnP	R	10181	10248	68	TGG			0
ND6	F	10266	10772	507		ATA	TAA	17
CytB	F	10781	11920	1140		ATG	TAA	8
trnS2	F	11945	12012	68	TGA			24
ND1	R	12031	12957	927		ATT	TAA	18
trnL1	R	12976	13043	68	TAG			18
rrnL	R	13082	14390	1309				38
trnV	R	14421	14485	65	TAC			30
rrnS	R	14484	15269	786				-2
trnI	F	16687	16753	67	GAT			1417

Table S3. Annotation of the mitogenome of *Dryocoetes hectographus.*

Gene	Direction	Start Position	Stop Position	Length	Anticodon	Start Codon	Stop Codon	IGS
trnQ	R	1	69	69	CAT			
trnM	F	69	139	71	TTG			-1
ND2	F	113	1132	1020		ATG	TAA	-27
trnW	F	1132	1197	66	TCA			-1
trnC	R	1197	1258	62	GCA			-1
trnY	R	1262	1325	64	GTA			3
COI	F	1318	2865	1548		ATT	TAA	-8
trnL2	F	2861	2925	65	TAA			-5
COII	F	2926	3612	687		ATT	TAA	0
trnK	F	3613	3683	71	CTT			0
trnD	F	3684	3751	68	GTC			0
ATP8	F	3752	3907	156		ATT	TAG	0
ATP6	F	3901	4572	672		ATG	TAA	-7
COIII	F	4573	5355	783		ATG	TAA	0
trnG	F	5362	5424	63	TCC			6
ND3	F	5425	5778	354		ATA	TAG	0
trnA	F	5777	5838	62	TGC			-2
trnR	F	5840	5907	68	TCG			1
trnN	F	5909	5972	64	GTT			1
trnS1	F	5973	6040	68	GCT			0
trnE	F	6041	6103	63	TTC			0
trnF	R	6127	6189	63	GAA			23
ND5	R	6143	7858	1716		ATT	TAG	-47
trnH	R	7898	7963	66	GTG			39
ND4	R	7944	9290	1347		ATG	TAA	-20
ND4L	R	9294	9587	294		ATG	TAG	3
trnT	F	9589	9652	64	TGT			1
trnP	R	9653	9715	63	TGG			0
ND6	F	9718	10227	510		ATT	TAA	2
CytB	F	10227	11369	1143		ATG	TAG	-1
trnS2	F	11368	11435	68	TGA			-2
ND1	R	11453	12379	927		ATA	TAA	17
trnL1	R	12405	12470	66	TAG			25
rrnL	R	12472	13756	1285				1
trnV	R	13758	13822	65	TAC			1
rrnS	R	13821	14596	776				-2
trnI	F	15432	15495	64	GAT			835

Table S4. Annotation of the mitogenome of *Polygraphus poligraphus.*

Gene	Direction	Start Position	Stop Position	Length	Anticodon	Start Codon	Stop Codon	IGS
trnI	F	1	63	63	GAT			
trnQ	R	1025	1092	68	CAT			961
trnM	F	1092	1160	69	TTG			-1
ND2	F	1161	2165	1005		ATT	TAA	0
trnW	F	2168	2235	68	TCA			2
trnC	R	2236	2302	67	GCA			0
trnY	R	2307	2371	65	GTA			4
COI	F	2364	3908	1545		ATT	TAA	-8
trnL2	F	3911	3973	63	TAA			2
COII	F	3995	4687	693		ATA	TAA	21
trnK	F	4653	4723	71	CTT			-35
trnD	F	4723	4786	64	GTC			-1
ATP8	F	4787	4945	159		ATT	TAG	0
ATP6	F	4939	5613	675		ATG	TAA	-7
COIII	F	5613	6398	786		ATG	TAA	-1
trnG	F	6405	6468	64	TCC			6
ND3	F	6469	6822	354		ATA	TAA	0
trnA	F	6831	6894	64	TGC			8
trnR	F	6895	6958	64	TCG			0
trnN	F	6960	7024	65	GTT			1
trnS1	F	7024	7083	60	GCT			-1
trnE	F	7085	7145	61	TTC			1
ND5	R	7155	8831	1677		ATG	TAA	9
trnH	R	8907	8973	67	GTG			75
ND4	R	8973	10301	1329		ATG	TAA	-1
ND4L	R	10295	10588	294		ATG	TAG	-7
trnT	F	10592	10656	65	TGT			3
trnP	R	10657	10720	64	TGG			0
ND6	F	10723	11229	507		ATG	TAA	2
CytB	F	11230	12369	1140		ATG	TAA	0
trnS2	F	12369	12434	66	TGA			-1
ND1	R	12444	13379	936		ATT	TAA	9
trnL1	R	13399	13465	67	TAG			19
rrnL	R	13426	14759	1334				-40
trnV	R	14748	14815	68	TAC			-12
rrnS	R	14815	15586	772				-1

Table S5. Annotation of the mitogenome of *Dendroctonus micans.*

Gene	Direction	Start Position	Stop Position	Length	Anticodon	Start Codon	Stop Codon	IGS
trnM	F	1	66	66	TTG			
ND2	F	88	1089	1002		ATT	TAA	21
trnW	F	1088	1151	64	TCA			-2
trnC	R	1151	1212	62	GCA			-1
trnY	R	1216	1278	63	GTA			3
COI	F	1271	2815	1545		ATT	TAA	-8
trnL2	F	2811	2875	65	TAA			-5
COII	F	2876	3559	684		ATT	TAA	0
trnK	F	3561	3631	71	CTT			1
trnD	F	3632	3695	64	GTC			0
ATP8	F	3696	3851	156		ATA	TAA	0
ATP6	F	3845	4516	672		ATG	TAA	-7
COIII	F	4522	5304	783		ATG	TAA	5
trnG	F	5311	5374	64	TCC			6
ND3	F	5375	5728	354		ATT	TAG	0
trnA	F	5727	5787	61	TGC			-2
trnR	F	5786	5852	67	TCG			-2
trnN	F	5851	5916	66	GTT			-2
trnS1	F	5917	5981	65	GCT			0
trnE	F	5981	6043	63	TTC			-1
trnF	R	6042	6104	63	GAA			-2
ND5	R	6061	7770	1710		ATT	TAG	-44
trnH	R	7813	7873	61	GTG			42
ND4	R	7857	9203	1347		ATG	TAA	-17
ND4L	R	9197	9493	297		ATG	TAA	-7
trnT	F	9497	9559	63	TGT			3
trnP	R	9560	9622	63	TGG			0
ND6	F	9625	10128	504		ATT	TAA	2
CytB	F	10128	11267	1140		ATG	TAA	-1
trnS2	F	11270	11334	65	TGA			2
ND1	R	11352	12281	930		ATA	TAG	17
trnL1	R	12301	12362	62	TAG			19
rrnL	R	12325	13629	1305				-38
trnV	R	13657	13722	66	TAC			27
rrnS	R	13720	14486	767				-3
trnI(gat)	F	16053	16117	65	GAT			1566
trnQ	R	16738	16807	70	CAT			620

Table S6 Annotation of the mitogenome of *Ips hauseri*

Gene	Direction	Start Position	Stop Position	Length	Anticodon	Start Codon	Stop Codon	IGS
trnQ	R	1	70	70	CAT			
trnM	F	123	193	71	TTG			52
ND2	F	194	1204	1011		ATA	TAA	0
trnW	F	1226	1292	67	TCA			21
trnC	R	1309	1373	65	GCA			16
trnY	R	1439	1506	68	GTA			65
COI	F	1470	3050	1581		ATT	TAA	-37
trnL2	F	3046	3113	68	TAA			-5
COII	F	3114	3794	681		ATT	TAA	0
trnK	F	3835	3905	71	CTT			40
trnD	F	3951	4019	69	GTC			45
ATP8	F	4020	4190	171		ATC	TAG	0
ATP6	F	4184	4864	681		ATG	TAA	-7
COIII	F	4876	5658	783		ATG	TAA	11
trnG	F	5714	5783	70	TCC			55
ND3	F	5784	6137	354		ATT	TAA	0
trnA	F	6150	6218	69	TGC			12
trnR	F	6317	6386	70	TCG			98
trnN	F	6418	6483	66	GTT			31
trnS1	F	6484	6551	68	GCT			0
trnE	F	6554	6621	68	TTC			2
trnF	R	6656	6723	68	GAA			34
ND5	R	6781	8488	1708		ATC	T-	57
trnH	R	8489	8557	69	GTG			0
ND4	R	8570	9907	1338		ATG	TAA	12
ND4L	R	9921	10214	294		ATA	TAG	13
trnT	F	10231	10297	67	TGT			16
trnP	R	10298	10365	68	TGG			0
ND6	F	10377	10874	498		ATT	TAA	11
CytB	F	10924	12063	1140		ATG	TAA	49
trnS2	F	12172	12240	69	TGA			108
ND1	R	12276	13212	937		TTG	T-	35
trnL1	R	13214	13283	70	TAG			1
rrnL	R	13300	14660	1361				16
trnV	R	14654	14720	67	TAC			-7
rrnS	R	14719	15516	798				-2

Table S7. Annotation of the mitogenome of *Ips subelongatus.*

Gene	Direction	Start Position	Stop Position	Length	Anticodon	Start Codon	Stop Codon	IGS
trnQ	R	1	71	71	CAT			
trnM	F	140	210	71	TTG			68
ND2	F	214	1221	1008		ATA	TAA	3
trnW	F	1223	1289	67	TCA			1
trnC	R	1296	1359	64	GCA			6
trnY	R	1373	1440	68	GTA			13
COI	F	1452	2999	1548		ATT	TAA	11
trnL2	F	3012	3079	68	TAA			12
COII	F	3080	3760	681		ATC	TAA	0
trnK	F	3804	3874	71	CTT			43
trnD	F	3880	3947	68	GTC			5
ATP8	F	3948	4121	174		ATT	TAG	0
ATP6	F	4115	4795	681		ATG	TAA	-7
COIII	F	4820	5602	783		ATG	TAA	24
trnG	F	5630	5700	71	TCC			27
ND3	F	5704	6054	351		ATC	TAA	3
trnA	F	6064	6128	65	TGC			9
trnR	F	6147	6215	69	TCG			18
trnN	F	6248	6314	67	GTT			32
trnS1	F	6315	6381	67	GCT			0
trnE	F	6384	6448	65	TTC			2
trnF	R	6456	6524	69	GAA			7
ND5	R	6534	8276	1743		ATT	TAA	9
trnH	R	8277	8348	72	GTG			0
ND4	R	8373	9710	1338		ATG	TAA	24
ND4L	R	9726	10019	294		ATA	TAA	15
trnT	F	10067	10133	67	TGT			47
trnP	R	10134	10199	66	TGG			0
ND6	F	10202	10711	510		ATT	TAA	2
CytB	F	10761	11897	1137		ATG	TAA	49
trnS2	F	11912	11981	70	TGA			14
ND1	R	12001	12948	948		TTG	TAA	19
trnL1	R	12950	13018	69	TAG			1
rrnL	R	13004	14367	1364				-15
trnV	R	14383	14448	66	TAC			15
rrnS	R	14447	15259	813				-2

Table S8. Annotation of the mitogenome of *Ips typographus.*

Gene	Direction	Start Position	Stop Position	Length	Anticodon	Start Codon	Stop Codon	IGS
trnQ	R	1	71	71	CAT			
trnM	F	89	160	72	TTG			17
ND2	F	164	1171	1008		ATA	TAA	3
trnW	F	1172	1239	68	TCA			0
trnC	R	1269	1338	70	GCA			29
trnY	R	1344	1410	67	GTA			5
COI	F	1439	3007	1569		ATT	TAA	28
trnL2	F	3031	3099	69	TAA			23
COII	F	3100	3780	681		ATC	TAA	0
trnK	F	3811	3882	72	CTT			30
trnD	F	3898	3965	68	GTC			15
ATP8	F	3966	4130	165		ATT	TAG	0
ATP6	F	4127	4804	678		ATA	TAA	-4
COIII	F	4810	5592	783		ATG	TAA	5
trnG	F	5618	5683	66	TCC			25
ND3	F	5687	6037	351		ATT	TAA	3
trnA	F	6049	6117	69	TGC			11
trnR	F	6139	6208	70	TCG			21
trnN	F	6241	6306	66	GTT			32
trnS1	F	6307	6374	68	GCT			0
trnE	F	6376	6445	70	TTC			1
trnF	R	6450	6520	71	GAA			4
ND5	R	6551	8293	1743		ATT	TAA	30
trnH	R	8294	8359	66	GTG			0
ND4	R	8392	9729	1338		ATG	TAA	32
ND4L	R	9736	10113	378		ATT	TAA	6
trnT	F	10050	10116	67	TGT			-64
trnP	R	10117	10183	67	TGG			0
ND6	F	10195	10692	498		ATT	TAA	11
CytB	F	10785	11927	1143		ATA	TAA	92
trnS2	F	12019	12088	70	TGA			91
ND1	R	12108	13055	948		TTG	TAA	19
trnL1	R	13057	13125	69	TAG			1
rrnL	R	13144	14509	1366				18
trnV	R	14502	14566	65	TAC			-8
rrnS	R	14565	15376	812				-2

Table S9. Annotation of the mitogenome of *Ips nitidus.*

Gene	Direction	Start Position	Stop Position	Length	Anticodon	Start Codon	Stop Codon	IGS
trnQ	R	1	71	71	CAT			
trnM	F	87	158	72	TTG			15
ND2	F	159	1169	1011		ATT	TAA	0
trnW	F	1170	1237	68	TCA			0
trnC	R	1263	1333	71	GCA			25
trnY	R	1342	1408	67	GTA			8
COI	F	1446	3002	1557		ATT	TAA	37
trnL2	F	3022	3090	69	TAA			19
COII	F	3091	3771	681		ATC	TAA	0
trnK	F	3802	3873	72	CTT			30
trnD	F	3890	3957	68	GTC			16
ATP8	F	3958	4122	165		ATT	TAG	0
ATP6	F	4119	4796	678		ATA	TAA	-4
COIII	F	4802	5584	783		ATG	TAA	5
trnG	F	5610	5675	66	TCC			25
ND3	F	5676	6029	354		ATT	TAA	0
trnA	F	6041	6109	69	TGC			11
trnR	F	6131	6200	70	TCG			21
trnN	F	6233	6298	66	GTT			32
trnS1	F	6299	6366	68	GCT			0
trnE	F	6368	6438	71	TTC			1
trnF	R	6443	6513	71	GAA			4
ND5	R	6535	8280	1746		ATC	TAA	21
trnH	R	8281	8346	66	GTG			0
ND4	R	8387	9724	1338		ATG	TAA	40
ND4L	R	9731	10018	288		ATT	TAG	6
trnT	F	10045	10111	67	TGT			26
trnP	R	10112	10178	67	TGG			0
ND6	F	10190	10687	498		ATT	TAA	11
CytB	F	10778	11920	1143		ATA	TAA	90
trnS2	F	12000	12069	70	TGA			79
ND1	R	12089	13036	948		TTG	TAA	19
trnL1	R	13038	13106	69	TAG			1
rrnL	R	13124	14486	1363				17
trnV	R	14480	14544	65	TAC			-7
rrnS	R	14543	15359	817				-2

Table S10. Investigation of nucleotide composition in the mitochondrial genomes of eight bark beetles.

Species	Length	T %	C %	A %	G %	A+T	C+G	AT-ske	GC-ske
Whole genome									
Orthotomicus erosus	16753	33.48	18.64	37.21	10.67	70.69	29.31	0.0528	–0.2722
Dryocoetes hectographus	15495	35.39	15.88	39.14	9.60	74.52	25.48	0.0503	–0.2467
Polygraphus poligraphus	15586	33.37	17.46	37.69	11.49	71.06	28.95	0.0608	–0.2458
Dendroctonus micans	16807	34.57	15.08	41.16	9.20	75.72	24.28	0.0870	–0.2421
Ips hauseri	15516	35.45	16.87	37.90	9.78	73.35	26.65	0.0335	–0.2662
Ips subelongatus	15259	35.92	15.80	38.79	9.50	74.70	25.30	0.0384	–0.2489
Ips typographus	15376	35.18	16.63	38.22	9.97	73.40	26.60	0.0414	–0.2501
Ips nitidus	15359	35.92	15.80	38.79	9.50	74.70	25.30	0.0384	–0.2489
Average (*Ips*)	15425	34.78	16.61	38.55	10.06	73.33	26.67	0.0514	–0.2463
PCGs									
Orthotomicus erosus	11162	40.09	17.31	27.78	14.82	67.87	32.13	–0.1814	–0.0775
Dryocoetes hectographus	11157	41.76	13.62	31.50	13.12	73.25	26.75	–0.1401	–0.0188
Polygraphus poligraphus	11091	40.24	17.05	28.78	13.93	69.02	30.98	–0.1660	–0.1007
Dendroctonus micans	11124	41.68	14.43	31.57	12.32	73.25	26.75	–0.1379	–0.0786
Ips hauseri	11177	41.06	15.95	29.14	13.85	70.20	29.80	–0.1698	–0.0705
Ips subelongatus	11196	42.43	14.22	30.44	12.92	72.87	27.13	–0.1645	–0.0481
Ips typographus	11196	41.94	14.83	30.16	13.07	72.10	27.90	–0.1634	–0.0629
Ips nitidus	11190	41.36	15.39	29.85	13.40	71.21	28.79	–0.1616	–0.0689
Average (*Ips*)	11162	41.32	15.35	29.90	13.43	71.22	28.78	–0.1606	–0.0658
rRNA									
Orthotomicus erosus	2095	39.62	7.64	36.13	16.61	75.75	24.25	–0.0460	0.3701
Dryocoetes hectographus	2061	37.99	7.96	36.68	17.37	74.67	25.33	–0.0175	0.3716
Polygraphus poligraphus	2016	39.08	8.02	35.80	17.09	74.88	25.12	–0.0438	0.3611
Dendroctonus micans	2072	43.29	6.71	36.34	13.66	79.63	20.37	–0.0873	0.3412
Ips hauseri	2159	39.97	6.67	38.72	14.64	78.69	21.31	–0.0159	0.3739
Ips subelongatus	2177	40.24	6.66	38.31	14.79	78.55	21.45	–0.0246	0.3790
Ips typographus	2178	40.22	6.80	38.06	14.92	78.28	21.72	–0.0276	0.3742
Ips nitidus	2180	39.86	7.16	37.98	15.00	77.84	22.16	–0.0242	0.3540
Average (*Ips*)	2117	40.03	7.20	37.25	15.51	77.29	22.71	–0.0358	0.3656
tRNA									
Orthotomicus erosus	1480	35.34	11.62	39.73	13.31	75.07	24.93	0.0585	0.0678
Dryocoetes hectographus	1443	37.35	9.70	39.29	13.65	76.65	23.35	0.0253	0.1691
Polygraphus poligraphus	1443	35.47	12.67	36.85	15.00	72.32	27.68	0.0191	0.0842
Dendroctonus micans	1419	37.56	10.50	39.18	12.76	76.74	23.26	0.0211	0.0970
Ips hauseri	1438	37.48	9.94	38.73	13.84	76.22	23.78	0.0164	0.1637
Ips subelongatus	1431	37.32	9.92	40.39	12.37	77.71	22.29	0.0396	0.1097
Ips typographus	1441	35.60	10.96	40.67	12.77	76.27	23.73	0.0664	0.0760
Ips nitidus	1443	36.17	10.46	40.12	13.24	76.30	23.70	0.0518	0.1170
Average (*Ips*)	1442	36.54	10.72	39.37	13.37	75.91	24.09	0.0373	0.1106

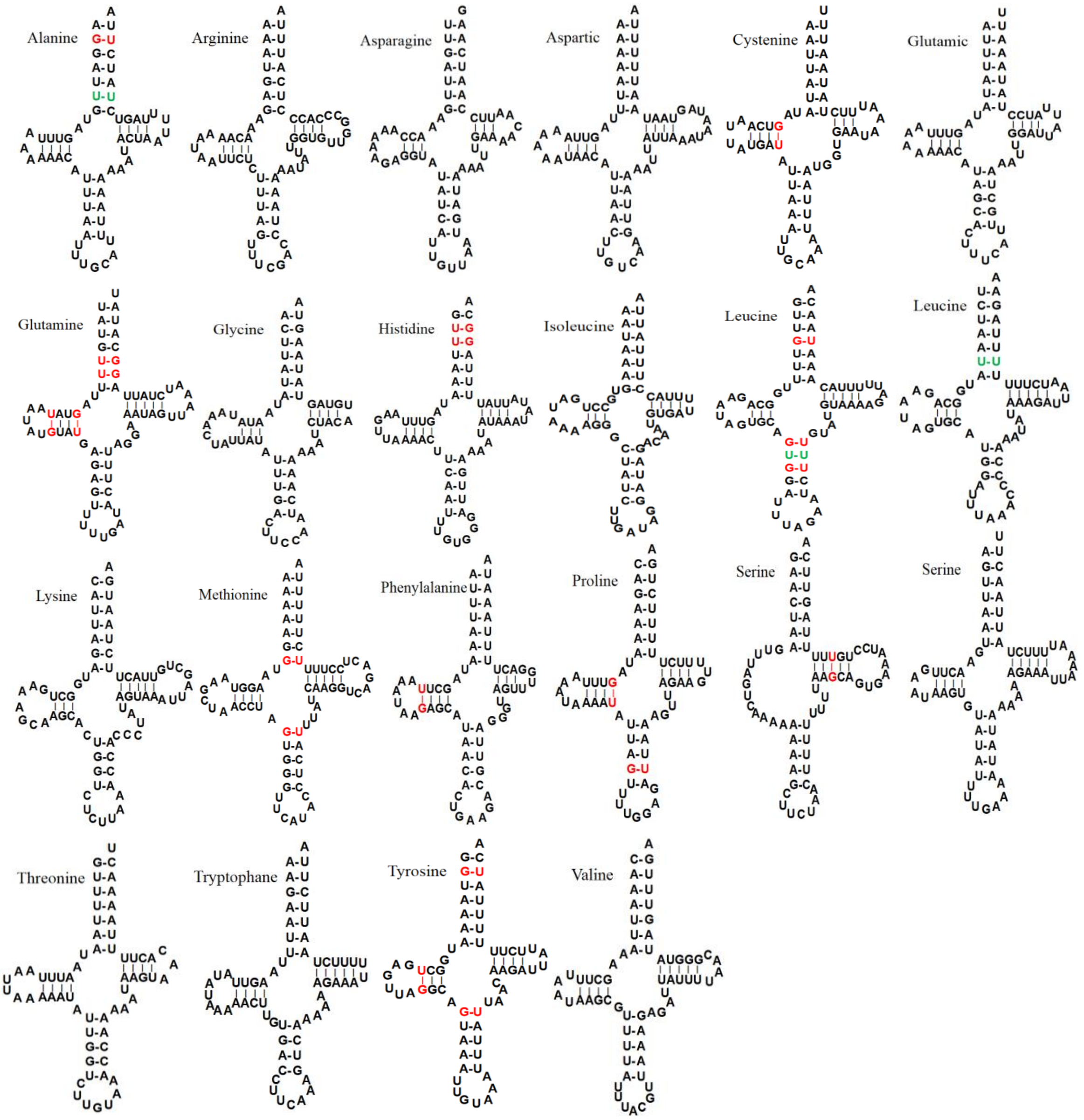

Figure S1. Secondary structures for tRNA genes from the mtDNA of *Dryocoetes hectographus*

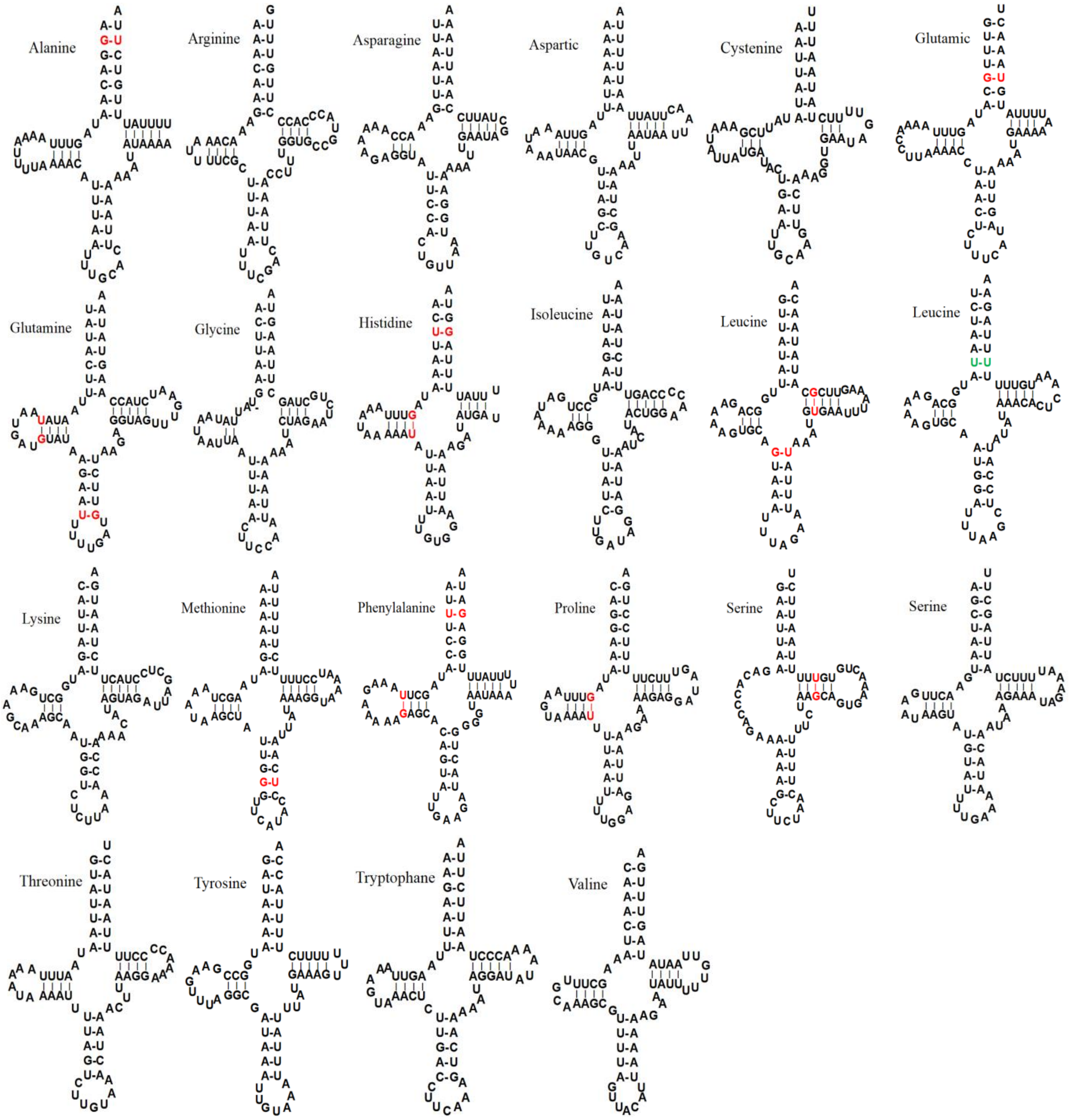

Figure S2. Secondary structures for tRNA genes from the mtDNA of *Orthotomicus erosus*

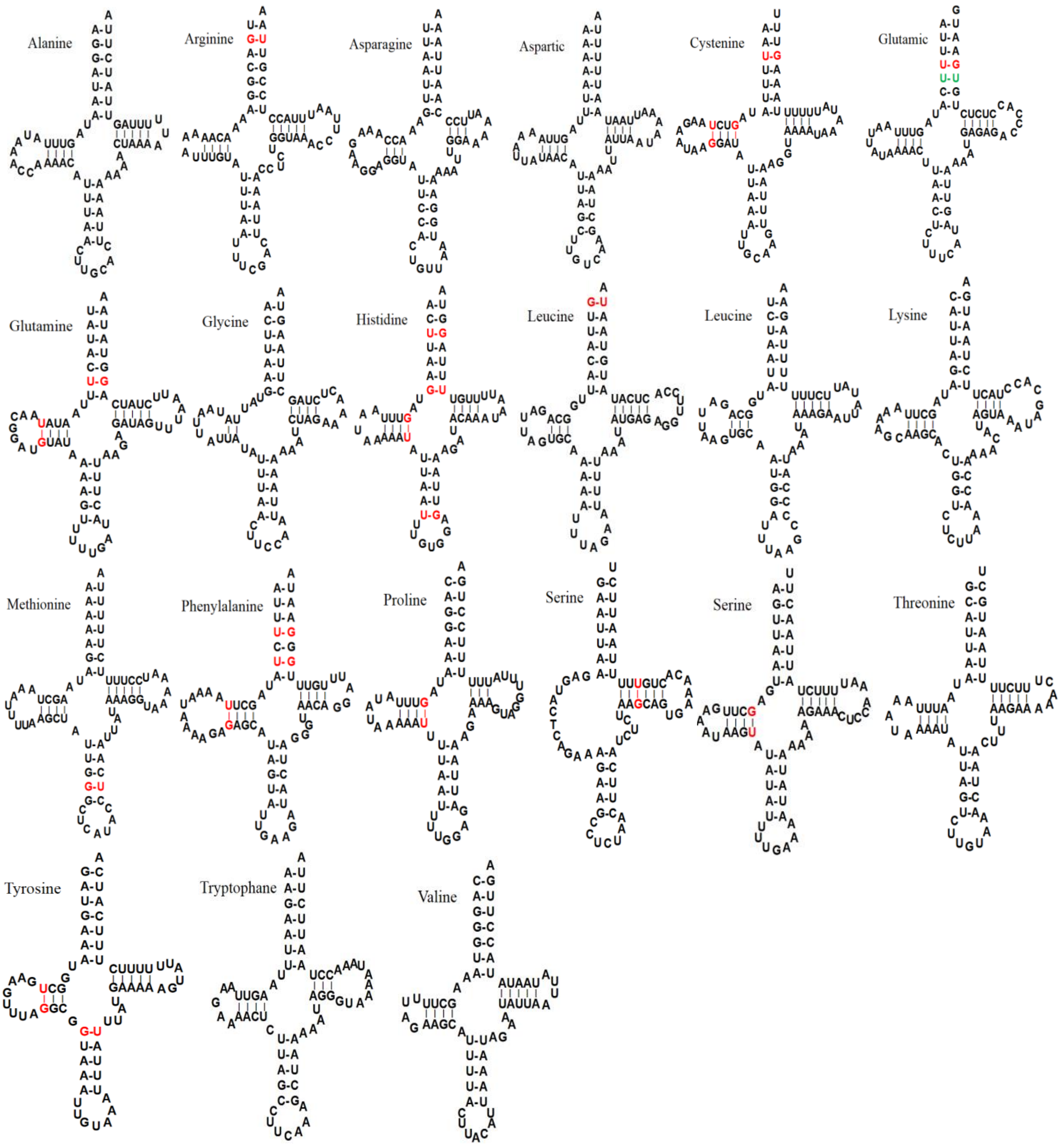

Figure S3. Secondary structures for tRNA genes from the mtDNA of *Ips typographus*

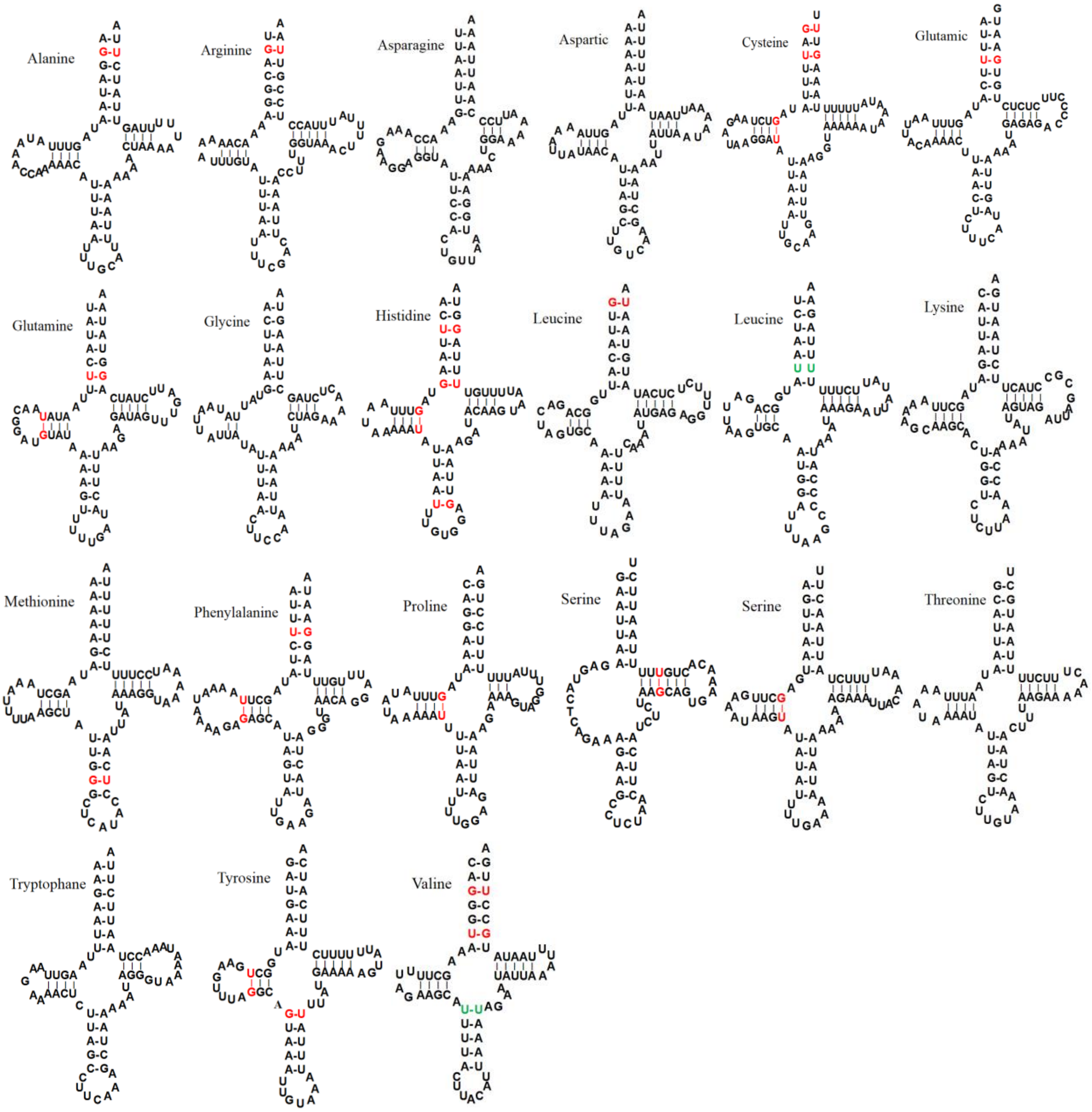

Figure S4. Secondary structures for tRNA genes from the mtDNA of *Ips nitidus*

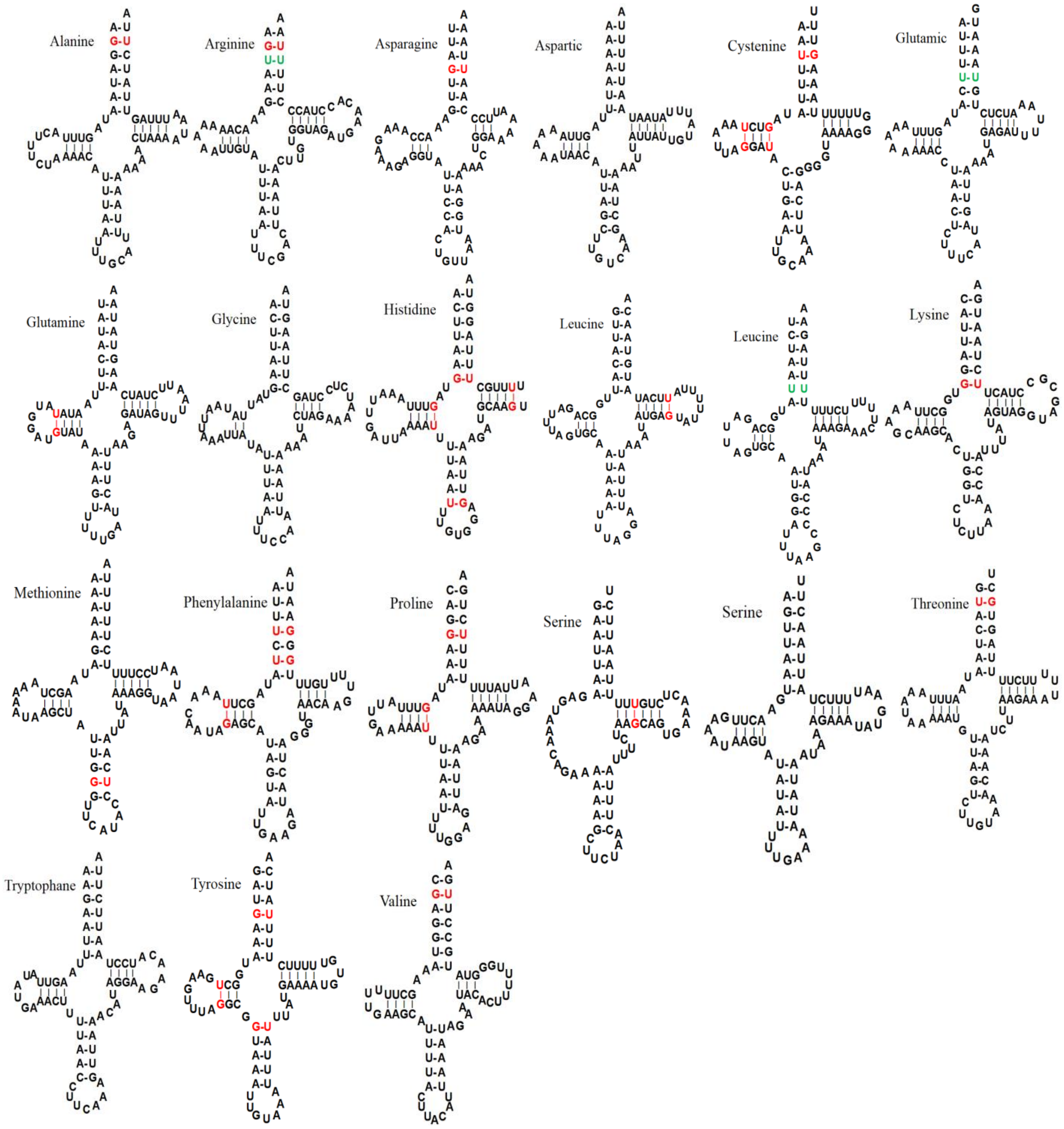

Figure S5. Secondary structures for tRNA genes from the mtDNA of *Ips hauseri*

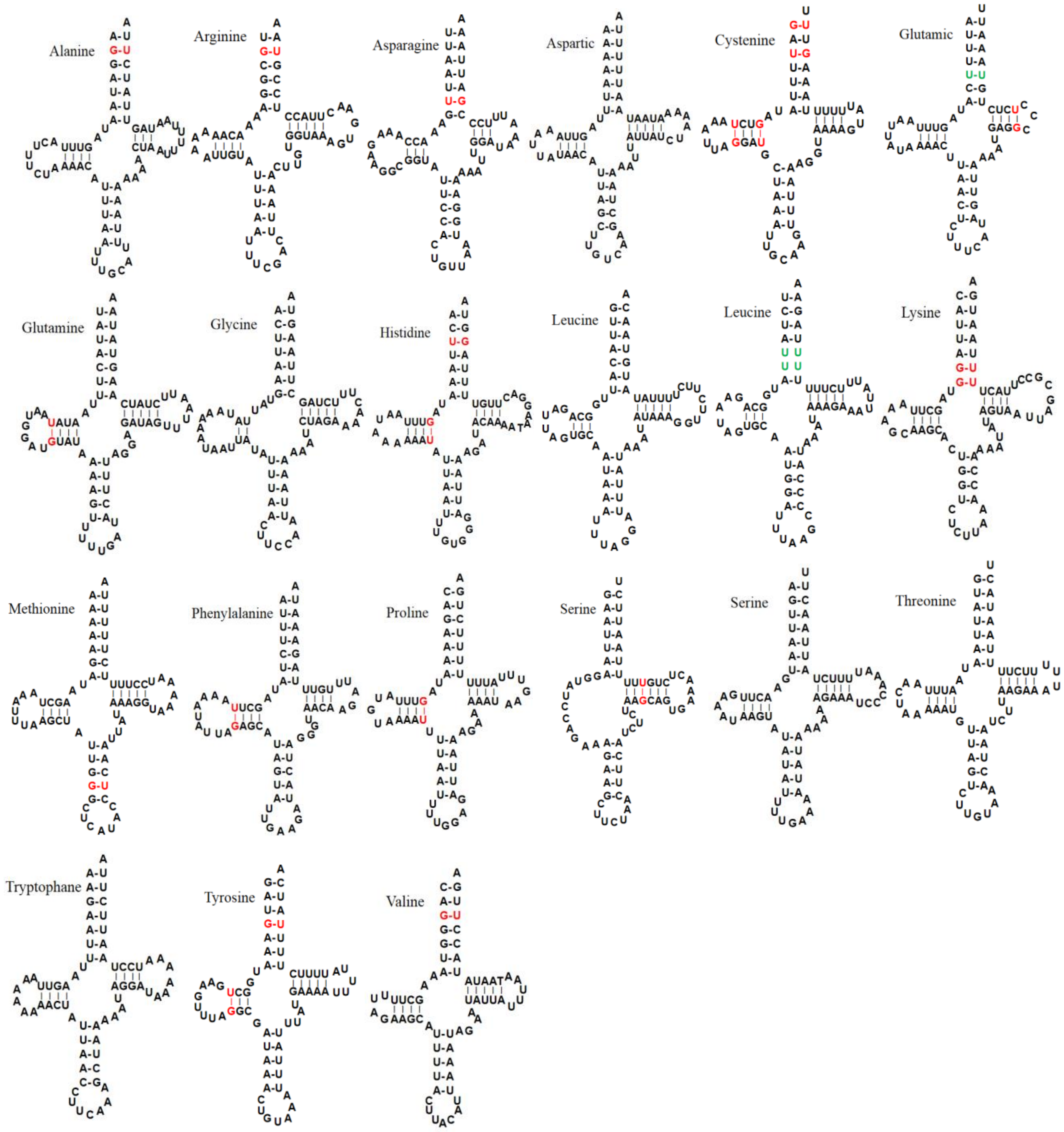

Figure S6. Secondary structures for tRNA genes from the mtDNA of *Ips subelongatus*

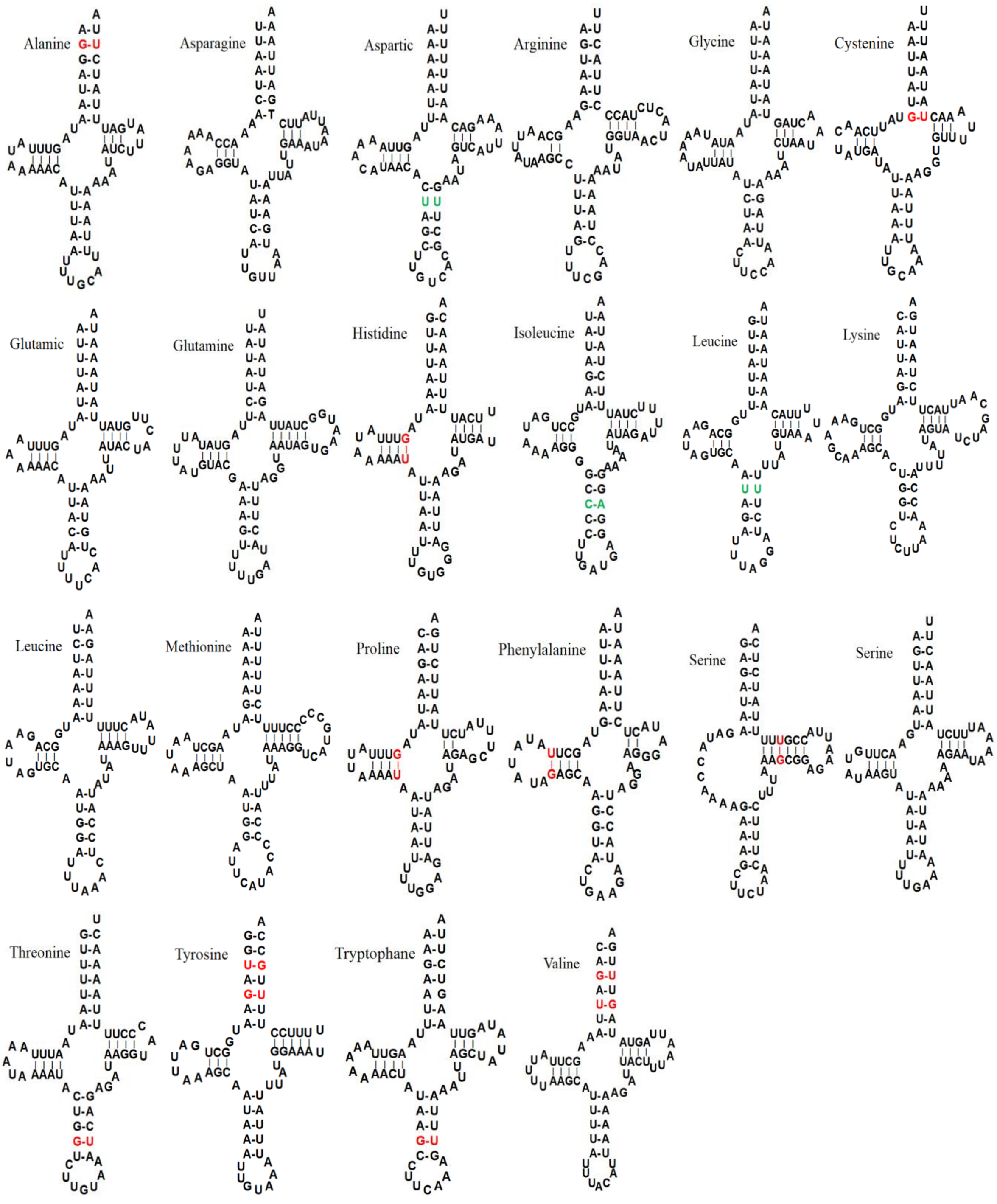

Figure S7. Secondary structures for tRNA genes from the mtDNA of *Dendroctonus micans*

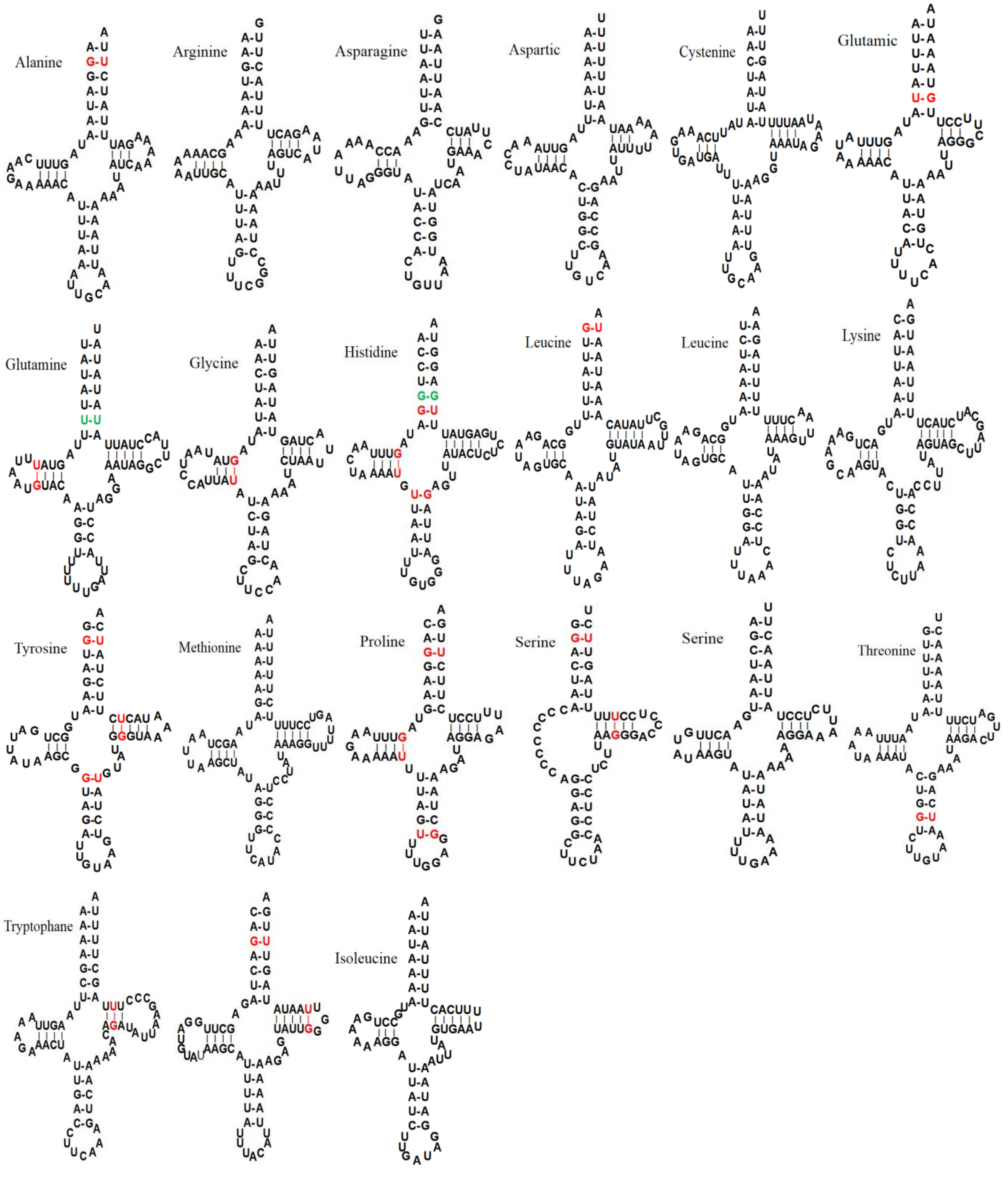

Figure S8. Secondary structures for tRNA genes from the mtDNA of *Polygraphus poligraphus*

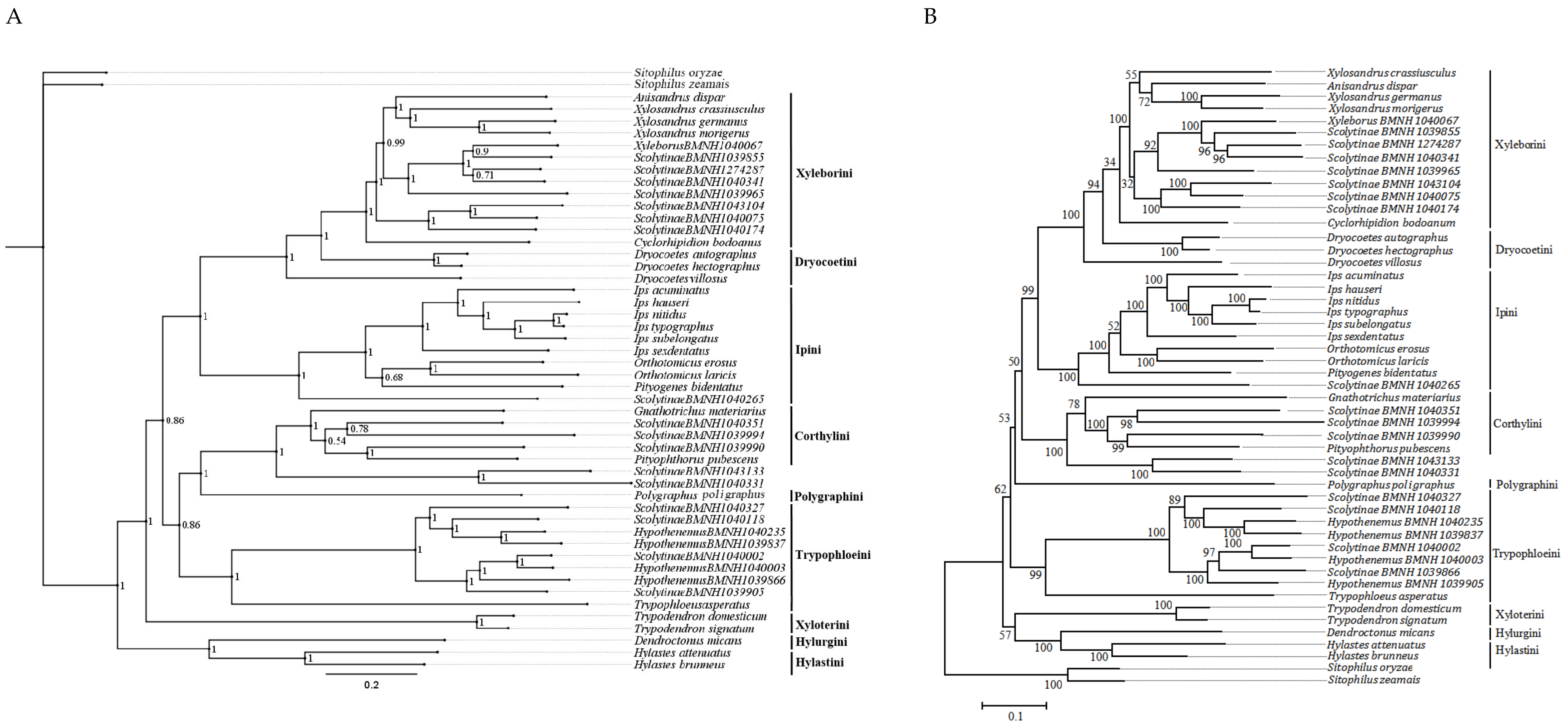

Figure S9. Phylogenetic tree inferred from mitogenomes of subfamily Scolytinae using Bayesian inference method (A) and maximum likelihood method (B). Values at nodes indicate the bootstrap values of BI and ML tree respectively.
